# Advances in methods for characterising dietary patterns: a scoping review

**DOI:** 10.1017/S0007114524002587

**Published:** 2025-04-14

**Authors:** Joy M. Hutchinson, Amanda Raffoul, Alexandra Pepetone, Lesley Andrade, Tabitha E. Williams, Sarah A. McNaughton, Rebecca M. Leech, Jill Reedy, Marissa M. Shams-White, Jennifer E. Vena, Kevin W. Dodd, Lisa M. Bodnar, Benoît Lamarche, Michael P. Wallace, Megan Deitchler, Sanaa Hussain, Sharon I. Kirkpatrick

**Affiliations:** 1 School of Public Health Sciences, University of Waterloo, Waterloo, ON, Canada; 2 Department of Nutritional Sciences, University of Toronto, Toronto, ON, Canada; 3 Health and Well-Being Centre for Research Innovation, School of Human Movement and Nutrition Sciences, University of Queensland, St. Lucia, QLD, Australia; 4 Institute for Physical Activity and Nutrition, School of Exercise and Nutrition Sciences, Deakin University, Geelong, VIC, Australia; 5 National Cancer Institute, National Institutes of Health, Bethesda, MD, USA; 6 Population Science Department, American Cancer Society, Washington, DC, USA; 7 Division of Cancer Control and Population Sciences, National Cancer Institute, Bethesda, MD, USA; 8 Alberta’s Tomorrow Project, Alberta Health Services, Edmonton, AB, Canada; 9 Division of Cancer Prevention, National Cancer Institute, Bethesda, MD, USA; 10 School of Public Health, University of Pittsburgh, Pittsburgh, PA, USA; 11 Centre Nutrition, santé et société (NUTRISS), Institut sur la nutrition et les aliments fonctionnels (INAF), Université Laval, Québec City, QC, Canada; 12 Department of Statistics and Actuarial Science, University of Waterloo, Waterloo, ON, Canada; 13 Intake – Center for Dietary Assessment, FHI Solutions, Washington, DC, USA

**Keywords:** Dietary patterns, Scoping review, Novel methods, Machine learning, Latent class analysis, Diet quality, Health outcomes

## Abstract

There is a growing focus on understanding the complexity of dietary patterns and how they relate to health and other factors. Approaches that have not traditionally been applied to characterise dietary patterns, such as latent class analysis and machine learning algorithms, may offer opportunities to characterise dietary patterns in greater depth than previously considered. However, there has not been a formal examination of how this wide range of approaches has been applied to characterise dietary patterns. This scoping review synthesised literature from 2005 to 2022 applying methods not traditionally used to characterise dietary patterns, referred to as novel methods. MEDLINE, CINAHL and Scopus were searched using keywords including latent class analysis, machine learning and least absolute shrinkage and selection operator. Of 5274 records identified, 24 met the inclusion criteria. Twelve of twenty-four articles were published since 2020. Studies were conducted across seventeen countries. Nine studies used approaches with applications in machine learning, such as classification models, neural networks and probabilistic graphical models, to identify dietary patterns. The remaining studies applied methods such as latent class analysis, mutual information and treelet transform. Fourteen studies assessed associations between dietary patterns characterised using novel methods and health outcomes, including cancer, cardiovascular disease and asthma. There was wide variation in the methods applied to characterise dietary patterns and in how these methods were described. The extension of reporting guidelines and quality appraisal tools relevant to nutrition research to consider specific features of novel methods may facilitate consistent reporting and enable synthesis to inform policies and programs.

## Graphical abstract

**Figure:**
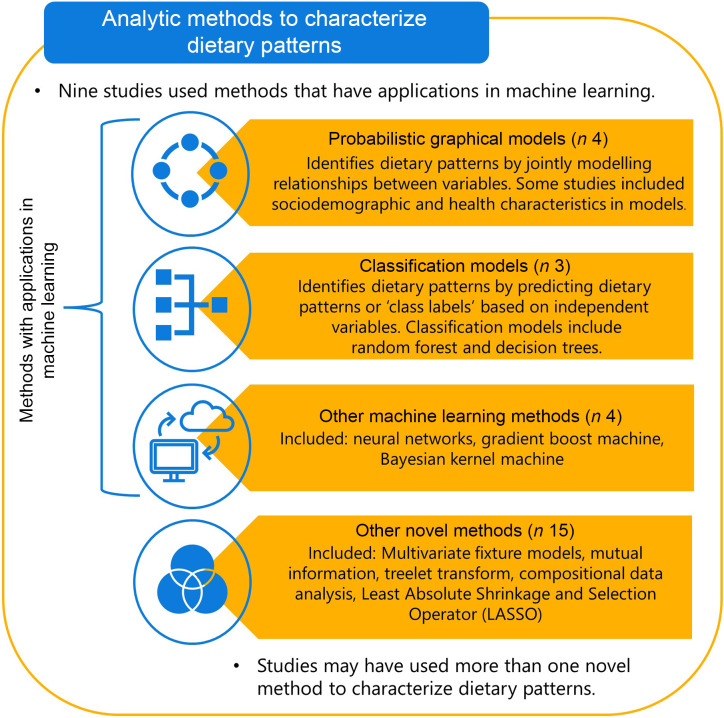

Dietary intake is among the top risk factors for chronic diseases^(1,2)^. Research examining dietary intake has historically focused on single foods, nutrients or other dietary constituents^(3)^. As the focus of public health nutrition shifted from the prevention of deficiency to the prevention of chronic diseases, research likewise shifted towards the examination of dietary patterns, aiming to capture how foods and beverages are consumed in real life^(3–5)^. Humans typically do not consume foods or nutrients on their own, but in the context of a broader dietary pattern^(3,4)^. Accordingly, food-based dietary guidelines are now typically focused on patterns of intake rather than single dietary components^(6)^. It is likely the synergistic and antagonistic relationships among the multiple foods, beverages and other dietary components that humans consume that influence health rather than individual components^(4)^. In addition to this multidimensionality, dietary patterns are dynamic, changing from meal to meal, day to day and across the life course^(4,7)^. Further, dietary patterns are shaped by culture, social position and other contextual factors^(8,9)^. However, incorporating the domains of multidimensionality, dynamism and contextual factors into dietary patterns analysis is a difficult task.

Traditional approaches to identify dietary patterns, including ‘a priori’ and ‘a posteriori’ approaches, are useful for understanding overall dietary patterns or the diet quality of populations and population subgroups^(10)^. For example, ‘a priori’ methods like the Healthy Eating Index-2020 and the Healthy Eating Food Index-2019 are generally investigator driven^(11,12)^ and consider multiple components such as fruits and vegetables and whole grains as inputs, but typically compress the multidimensional construct of dietary patterns to a single unidimensional score reflecting overall diet quality^(13,14)^. ‘A posteriori’ approaches are data-driven and have also been widely used to identify dietary patterns. Commonly applied data-driven approaches include clustering methods (e.g., k-means, Ward’s method), principal component analysis and factor analysis, providing opportunities to identify dietary patterns through statistical modelling or clustering algorithms rather than relying on researcher hypotheses^(15)^. These approaches compress dietary components to key food groupings typically expressed as single scores^(10,16)^. By reducing the dimensionality of dietary patterns, these methods are limited in their ability to explain the wide variation in dietary intakes^(4)^. Methods employed to traditionally characterise dietary patterns using ‘a priori’ and ‘a posteriori’ approaches thus address multidimensionality to some extent, but do not allow for explorations of dietary patterns in their totality because they miss potential synergistic or antagonistic associations among dietary components^(4,14,17)^.

Novel methods that have not traditionally been used to identify dietary patterns, such as probabilistic graphical modelling, latent class analysis and machine learning algorithms (e.g., random forest, neural networks), may capture complexities like dietary synergy. There is no clear delineation between traditional and novel methods, and specifically defining what is novel is challenging given it naturally implies an evolution of methods. Nonetheless, there is a growing interest among nutrition researchers in the application of methods that have not typically been used to capture dietary complexity, with these methods often centred in machine learning^(18)^. To date, there have been perspectives and narrative reviews on the application of machine learning in nutrition^(19–21)^, and a recent systematic review of studies that applied machine learning approaches to assess food consumption^(22)^. However, there has not been an assessment of studies applying novel methods to characterise dietary patterns. Given the rapid adoption of these methods within the field of health^(23–26)^, it is increasingly important for researchers to have a basic understanding of available methods and how they are being applied in the field. This will facilitate the synthesis of evidence from a range of methodological inputs to inform food-based dietary guidelines and other policies and programs that promote health. The objective of this scoping review was therefore to describe the use of novel methods not traditionally used to characterise dietary patterns in the published literature.

## Methods

The review was conducted in accordance with the JBI Manual for Evidence Synthesis^(27)^, which was developed using the Arksey and O’Malley framework^(28)^. Reporting follows the Preferred Reporting Items for Systematic Reviews and Meta-Analyses extension for Scoping Reviews^(29)^.

### Defining novel methods

The novel methods considered were based on a preliminary search of the literature and the expertise of the research team and included systems methods (e.g., agent-based modelling, system dynamics), least absolute shrinkage and selection operator, machine learning algorithms, copulas and data-driven statistical modelling approaches (e.g., treelet transformations, principal balances and coordinates). Novel methods could also include those that have been used previously in nutrition research if applied in new ways to characterise dietary patterns (e.g., linear programming used to model a modified dietary pattern rather than to test scenarios). Methods that were not considered to be novel were those that have been applied to assess dietary patterns in numerous studies and have been considered by prior reviews and commentaries^(2,10,30)^, including regression, ‘a priori’ approaches such as investigator-driven indices, and routinely used data-driven approaches, including factor analysis and cluster analysis^(10,31)^.

### Identifying relevant studies

Articles were eligible for inclusion if they were: a primary research article; focused on dietary intake as an exposure or outcome, including examination of dietary patterns (i.e., multiple dietary components in combination rather than single nutrients, foods or other dietary components); used at least one or more novel methods, as described above, to characterise dietary patterns; were published in English; and focused on humans. Ineligible studies included those focused on individual foods or human milk rather than dietary patterns and commentaries and reviews.

Searches of three research databases, MEDLINE (via PubMed), the Cumulative Index to Nursing and Allied Health Literature and Scopus, were conducted in March 2022. These health-focused, specialised and multidisciplinary databases were selected based on consultation with a research librarian (JS) to ensure a range of possibly relevant study types were included. The search strategies were developed in consultation with the research librarian using keywords and subject headings to capture diet-related constructs (e.g., dietary intake, patterns, recommendations, feeding behaviour, food habits) and novel methods to characterise dietary patterns (e.g., machine learning, network science and system dynamics model). No date limits were applied to the searches, and articles were included until the end of the search in March 2022. The search strategies for MEDLINE, CINAHL and Scopus are available in online Supplementary File 1.

### Study selection

Two independent reviewers (two of AP, AR, SH, SIK) screened each record at the title and abstract and full-text screening stages using Covidence^(32)^, with one consistent reviewer (AP) participating throughout the entire process. At the title and abstract screening stage, an initial pilot screening (twenty-five records) generated 100 % agreement (AR and AP) and 92 % agreement (AP and SH). A second pilot screening (100 records) generated 91 % agreement (AR and AP) and 93 % agreement (AP and SH). When applicable, discrepancies were discussed by reviewers and if needed deferred to a third reviewer (SIK) for decision. Following pilot screening, the reviewers independently reviewed the remaining articles (96 % agreement, Kappa = 0·83).

The reviewers were intentionally liberal during the title and abstract screening stage because of the breadth of possible novel methods. This required iteratively revisiting the inclusion criteria. For example, reduced rank regression was initially considered to be novel but was found to be prevalent in the literature based on title and abstract screening and was excluded during full-text review. Further, articles that used ‘a posteriori’ methods to identify dietary intake but did not specify the exact method in the title or abstract were included for full-text review.

Pilot screening of full-text reviews (fifty records) generated 82 % agreement (AR and AP) and 96 % agreement (AP and SIK); after discrepancies were discussed, two reviewers independently screened the remaining full-text articles (93 % agreement, Kappa = 0·60). The high agreement between reviewers but relatively low Cohen’s Kappa is described as Cohen’s paradox, with a larger number of studies excluded than included^(33–35)^.

### Data extraction

Data extraction was completed by JMH and TEW using a pre-specified Excel template, with all extracted data subsequently verified by LA. Data extraction fields (online Supplementary File 2) included information pertaining to authorship, study title, journal, year of publication, funding source, contextual details (e.g., study location), sample size and participant characteristics (e.g., age). Details relating to study methods (e.g., analysis input variables, measurement of dietary intake and analytic approaches) and results (e.g., findings related to dietary patterns and if applicable, health risk and outcomes) were also extracted.

## Results

### Summary of search

**Figure 1.**
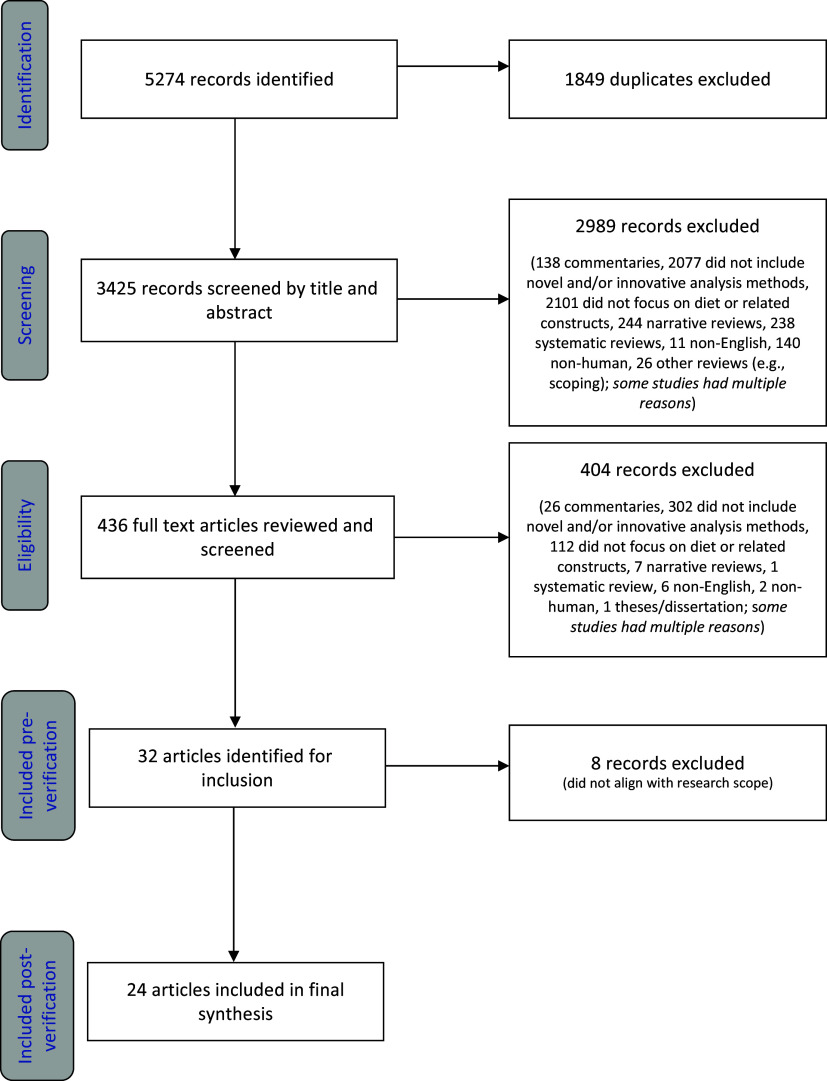
PRISMA diagram illustrating the screening process for a scoping review exploring innovative methods for the analysis of dietary intake data and characterisation of dietary patterns. PRISMA, Preferred Reporting Items for Systematic Reviews and Meta-Analyses.

A total of 5274 unique articles were identified after removing duplicates. Of these, 436 were identified as potentially relevant based on the title and abstract review and underwent full-text screening (Figure 1). Studies excluded during full-text screening included those that did not include methods defined as novel, those that did not focus on dietary patterns, commentaries, narrative reviews, systematic reviews, studies that were not published in English, studies that were not conducted with humans and theses/dissertations. A final pool of twenty-four articles describing twenty-four unique studies met the inclusion criteria.

### Characteristics of included studies

Across the twenty-four included studies, data from seventeen countries were represented (Table 1). Half of the studies were published between 2005 and 2019^(36–47)^, and the remaining twelve were published between 2020 and March 2022^(48–59)^. Three studies used data from subsets of the European Prospective Investigation into Cancer and Nutrition^(36,45,46)^, two studies used waves of data from the National Health and Nutrition Examination Survey^(54,58)^ and two studies used data from the ELSA-Brasil cohort study (Table 2)^(42,57)^. Sample sizes ranged from 250 to over 73 000 participants. Nineteen studies were conducted using data from cohort or cross-sectional studies, and five studies applied a case–control design.

**Table 1.** Study characteristics across included studies applying novel methods to characterise dietary patterns

Characteristic	Number of articles
Country of study population^*^	
Austria	1
Brazil	2
China	2
Finland	1
France	3
Germany	3
Italy	1
Mexico	1
Netherlands	1
Northern Ireland	1
Poland	1
Portugal	3
Republic of Ireland	2
South Korea	1
Spain	1
Switzerland	1
USA	3
Year published	
2005–2009	2
2010–2014	3
2015–2019	7
2020–2022	12
Included health risk or outcomes	
Health risk/outcome (cross-sectional)	8
Health outcome (longitudinal)	6
No	10

* Some studies included more than one country.

**Table 2.** Characteristics of studies (*n* 24) identified in a scoping review of novel analytic methods to characterise dietary patterns

Authors, date, country(s)	Study aims	*n*	Study design and data source	Participant characteristics	Diet assessment method(s)
Affret *et al.*, 2017, France^(41)^	To characterise the relationship of dietary patterns with socio-economic factors	73 031	Cohort; E3N cohort^†^	Adults in France, females only, mean age 52·9 years	FFQ^‡^
Bezerra *et al.*, 2018, Brazil^(42)^	To examine how dietary patterns differ among generations in a Brazilian cohort	15 069	Cross-sectional analysis of cohort; ELSA-Brasil cohort^§^	Adults in Brazil, aged 35–74 years	FFQ
Biesbroek *et al.*, 2015, Netherlands^(36)^	To compare if two methods (traditional *v*. novel) derived differential dietary patterns and associations with health risk	34 644	Cohort; EPIC^||^ Prospect and EPIC MORGEN	Adults in the Netherlands, male and female, aged 20–70 years	FFQ
Dalmartello *et al.*, 2020, Italy^(48)^	To identify dietary patterns associated with oropharyngeal cancer	3438 (946 cases, 2492 controls)	Case–control; Unnamed study	Adults in Italy, admitted to major hospitals, 19–82 years	FFQ
De Almeida *et al.*, 2022, Brazil^(57)^	To investigate how a variety of approaches identify dietary patterns in the same dataset	12 816	Cohort; ELSA-Brasil cohort	Adults in Brazil, aged 35–74 years	FFQ
Farmer *et al.*, 2020, USA^(58)^	To explore how latent class analysis groups individuals into dietary patterns	11 481	Repeat cross-sectional; NHANES^¶^ 2007–2010	Adults in the USA, male and female, ≥ 19 years	Up to two 24HDRs^**^ (per day)
Fonseca *et al.*, 2012, Portugal^(37)^	To investigate the association between dietary patterns and metabolic syndrome	2167	Cross-sectional analysis of cohort study; EPIPorto cohort	Adults in Portugal, male and female, age not specified	FFQ
Harrington *et al.*, 2014, Republic of Ireland^(43)^	To investigate changes in dietary patterns across 10 years among Irish adults	925 (baseline) 320 (follow-up)	Cohort; Cork and Kerry Diabetes and Heart Disease Study	Adults in the Republic of Ireland, 50–69 years	FFQ
Hearty & Gibney, 2008, Republic of Ireland & Northern Ireland^(44)^	To compare the ability of two supervised machine learning techniques to predict diet quality based on dietary patterns	1379	Cross-sectional; North-South Ireland Food Consumption Survey	Adults in Northern Ireland and the Republic of Ireland, aged 18–64 years	7-day food diary
Hoang *et al.*, 2021, South Korea^(49)^	To describe interactions among dietary components, demographic characteristics and chronic disease risk factors	7423	Cross-sectional analysis of cohort study; Cancer Screening Examination Cohort	Adults in South Korea, 30–70 years	FFQ
Hose *et al.*, 2021, Austria, Finland, France, Germany, Switzerland^(50)^	To explore how feeding patterns across the first year of life relate to asthma in school-aged children	1246	Cohort; PASTURE^††^ Birth Cohort and LUKAS2^‡‡^ Cohort	Children in 5 European countries, birth cohort	Food diaries (weekly and monthly), dietary screener (monthly)
Iqbal *et al.*, 2016, Germany^(45)^	To identify dietary patterns among a German adult population	27 120	Cross-sectional analysis of cohort; EPIC Potsdam	Adults in Germany, male and female, aged 35–65 years	FFQ
Jamiolkowski *et al.*, 2005, Poland^(38)^	To identify dietary patterns and their associations with markers of health status	556	Cohort; Unnamed study	Adult males in Poland, age not specified	FFQ + 24HDR ‘24-hour consumption questionnaire’
Madeira *et al.*, 2021, Portugal^(51)^	To investigate associations between dietary patterns, malnutrition and BMI among older adults	849	Cross-sectional; PEN-3S Study^§§^	Adults in Portugal, ≥65 years	Two 24HDR (per day)
Oliveira *et al.*, 2011, Portugal^(39)^	To assess associations between a posteriori dietary patterns and health outcomes	3016 (820 cases, 2196 controls)	Case–control; Unnamed study	Adults in Portugal, male and female, aged ≥ 18 years	FFQ
Samieri *et al.*, 2020, France^(52)^	To model dietary patterns prior to the onset of dementia	1522	Case–control; 3-City Study	Adults in France, ≥ 65 years	FFQ
Schwedhelm *et al.*, 2018, Germany^(46)^	To describe dietary networks using machine learning methods across meals and usual patterns of eating	814	Cross-sectional analysis of cohort; EPIC Potsdam	Adults in Germany, male and female, average age 65·5 years	Up to three 24HDR (per day)
Schwedhelm *et al.*, 2021, USA^(59)^	To compare dietary patterns by meals among pregnant women with high and low diet quality	365	Cohort; Pregnancy Eating Attributes Study	Women receiving prenatal care in the USA, 18–44 years	Up to three 24HDRs, pooled
Shang *et al.*, 2020, China^(53)^	To characterise top dietary components that are associated with changes in cardiometabolic risk factors among children	5676	Cohort; Unnamed study	Children in China, 6–13 years	Three 24HDRs, assessed at baseline and one year. Average of recalls
Solans *et al.*, 2019, Spain^(47)^	To compare three compositional data analysis approaches to identify dietary patterns	3471	Case–control; Multi-Caso Control Spain	Adults in Spain, male and female, aged 20–85 years (controls only)	FFQ
Wright *et al.*, 2020, USA^(54)^	To examine how dietary patterns are associated with periodontitis	10 010	Repeat cross-sectional; NHANES 2009–2014	Adults in the USA, male and female, ≥ 30 years	Up to two 24HDRs Average of recalls
Wu *et al.*, 2019, Mexico^(40)^	To explore how maternal and adolescent diet are associated with pubertal tempo	250 (baseline) 222 (follow-up)	Cohort; ELEMENT cohort^||||^	Children in Mexico enrolled in a birth cohort	FFQ
Xia *et al.*, 2020, China^(55)^	To examine how dietary patterns differ between individuals with non-alcoholic fatty liver disease and controls	4086 (Split 50/50 between cases and controls)	Case-control; Tianjin Chronic Low-grade Systemic Inflammation and Health Cohort Study	Individuals in China	FFQ
Zhao *et al.*, 2021, USA^(56)^	To explore the relationship between dietary components, dietary patterns, and CVD	1928	Cross-sectional analysis of cohort; CARDIA cohort^¶¶^	Adults in the USA, 18–30 years	FFQ

† Etude Epidémiologique auprès de femmes de la Mutuelle Générale de l’Education Nationale.

‡ FFQ.

§ Brazilian Longitudinal Study of Adult Health.

|| European Prospective Investigation into Cancer and Nutrition.

¶ National Health and Nutrition Examination Survey.

** 24 h dietary recall.

†† Protection Against Allergy: Study in Rural Environments.

‡‡ Finnish, rural-suburban birth cohort.

§§ Portuguese Elderly and Nutritional Status Surveillance System.

|||| Early Life Exposure in Mexico to Environmental Toxicants.

¶¶ Coronary Artery Risk Development in Young Adults.

The majority (*n* 15) of studies used FFQ to assess dietary intake^(36,37,39–43,45,47–49,52,55–57)^. Six studies used 24-h recalls^(46,51,53,54,58,59)^, two studies used food records/diaries^(44,50)^ and one study used a FFQ and a 24-h recall^(38)^. Among the studies using 24-h recalls and records/diaries, one used data from a single recall that was combined with data from a FFQ^(38)^. The remaining studies including records or recalls averaged or combined data from two or more days of intake. Dietary input variables were created by selecting specific items of interest from questionnaires or condensing foods into groupings, ranging from nine to sixty-two food groupings^(36–59)^. Apart from averaging recalls or records, none of the included studies applied substantial efforts to mitigate measurement error present in dietary intake data. Several studies noted potential misreporting as a limitation, and five studies specifically noted that findings may have been influenced by measurement error present in self-reported dietary assessment instruments^(40,43,51,52,59)^.

### Novel methods applied to identify dietary patterns

The types of methods used and how they were implemented to identify dietary patterns varied widely (Table 3). Nine studies applied approaches that have applications in machine learning, including classification models, neural networks and probabilistic graphical models (Table 4)^(36,38,44–46,49,53,56,59)^. The earliest study included in this review was published in 2005 and applied neural networks to characterise dietary patterns^(38)^. Fifteen studies applied other novel methods, including latent class analysis, mutual information and treelet transform^(37,39–43,47,48,50–52,54,55,57,58)^. Two studies identified dietary patterns using more than one novel method^(44,53)^. Five studies included comparisons of different novel methods, though these were typically versions of the same model^(44,45,47,49,53)^. For example, Solans *et al.* compared three models for compositional data analysis and reported that the best-performing model incorporated both investigator- and data-driven methods^(47)^.

**Table 3.** Description of dietary patterns (*n* 24) identified in a scoping review of novel analytic methods to characterise dietary patterns

Authors, date, country(s)	Novel method(s) to analyse diet	Dietary input variables	Characterisation of dietary patterns	Consideration of health outcomes/risk	Consideration of socio-demographic characteristics^*^
***Studies that characterised dietary patterns to assess health outcomes/risk***					
Biesbroek *et al.*, 2015, Netherlands^(36)^	Random forest with classification tree analysis	Food items were grouped into thirty-five food groups based on Dutch standard food groups, represented as % of total energy.	Seven patterns were derived to explain cardiovascular disease risk factors: Prudent-like 1, Prudent-like 2, Traditional-like 1, Traditional-like 2, Traditional-like 3, Western-like 1, Western-like 2.	Investigated the association between dietary patterns and first occurrence of coronary artery disease, stroke, death from any cause through hazard ratios	All hazard ratios were adjusted for age and sex and one model additionally considered smoking status and education.
Dalmartello *et al.*, 2020, Italy^(48)^	Latent class analysis	Items from the FFQ were grouped into twenty-five food groups and daily intake (g/d) was estimated using standard portion sizes.	Latent class analysis was used to identify distinct dietary patterns: Western, Prudent, Lower consumers- combination, Higher consumers- combination.	Investigated associations of dietary patterns with risk of oropharyngeal cancer using OR.	Not stated how cases and controls were matched. OR were adjusted for age, sex, education, smoking status.
Fonseca *et al.*, 2012, Portugal^(37)^	Multivariate finite mixture models	Items from the FFQ were grouped into fourteen food groups based on similar nutritional characteristics.	Identified four dietary patterns among males and females. Males: healthy, fish, red meat and alcohol, intermediate. Females: healthy, low fruit and vegetable, red meat and alcohol, in transition to fast food.	Associations between metabolic syndrome and dietary patterns were assessed using crude and adjusted odds ratios	Identified dietary patterns by sex. OR were adjusted for age, education, smoking status.
Hoang *et al.*, 2021, South Korea^(49)^	Gaussian graphical models and mixed graphical models	Sixteen food groups, assessed as g/d, were included in graphical models. These food groups were also included in a graphical model as a single dietary score.	A network of intake was identified among the sizteen food groups, with edges representing conditional dependencies between food groups. Food groups that were not connected by an edge were conditionally independent.	Graphical models also included comorbidity factors, such as blood pressure or total cholesterol, to identify associations with dietary components.	Socio-demographic characteristics including age, sex, marital status, education and income were included in graphical models.
Hose *et al.*, 2021, Austria, Finland, France, Germany, Switzerland^(50)^	Latent class analysis	Included seventeen food items from nineteen-item screener that assessed time of introduction (screeners repeated at 2, 12, 18, 24, 36, 48, 60, 72 months of age) and frequency of consumption.	Four food introduction patterns were identified. Latent class 1 was characterised by daily fruit, vegetable and milk consumption; Latent class 2 was characterised by daily fruit, vegetable, and meat and cereal consumption, but less frequent milk consumption; Latent class 3 was characterised by slower introduction to foods, a diverse range of food items, but less than daily fruit and vegetable consumption; Latent class 4 was similar to Latent class 3 but with quicker introduction to foods.	Linear or logistic/multinomial regression was used to estimate associations between latent class analysis food introduction patterns, possible determinants and asthma outcomes.	Regression analyses adjusted for sex.
Jamiolkowski *et al.*, 2005, Poland^(38)^	Kohonen neural networks	Forty-one-item FFQ was included as input.	Three dietary patterns were identified: 1- high frequency of margarine, fried vegetable oil, low consumption of butter, lard, bacon, most frequent consumption of poultry, fresh fish, fruit; 2- high frequency butter, lard, giblets, smoked ‘second-rate’ meats, sweets, sugary beverages; 3- low frequency butter, margarine, milk, cheese.	Impact of dietary habits on blood serum lipids assessed using Kruskall–Wallis procedure.	No
Madeira *et al.*, 2021, Portugal^(51)^	Latent class transition model	Items were grouped from seventy-one food items to twenty-seven food groups.	Latent class transition models identified two dietary patterns, labelled as a protein-foods pattern and a Mediterranean pattern.	Linear regression was used to examine relationships between identified dietary patterns and nutritional status and BMI	Regression models adjusted for sex, age, education.
Oliveira *et al.*, 2011, Portugal^(39)^	Multivariate and univariate finite fixture models	Items from an eighty-two-item FFQ were grouped into fourteen food groups (g/d) based on nutritional similarities.	Distinct dietary patterns were identified among males and females using multivariate and univariate fixture models. Males: Healthy, Fish, Red meat and alcohol, intermediate intake. Females: Healthy, Low fruit and vegetables, red meat and alcohol, in transition to fast food.	Logistic regression was used to examine the association between dietary patterns and acute myocardial infarction (with and without confounders)	Identified dietary patterns by sex. Multivariate models included age, education, smoking status as confounders.
Samieri *et al.*, 2020, France^(52)^	Mutual information	148-item FFQ grouped into 62 dietary variables.	A mutual information matrix was computed to estimate the network relationships between dietary components. Networks of intake were estimated among cases and controls. The case network showed one mainly connected network of foods, whereas the controls network was represented by several disconnected subnetworks.	Two mutual information networks were developed and compared – one for dementia cases and one for controls. A third network representing the differences between the cases’ and controls’ dietary patterns was developed.	Nested case-control. Matched cases and controls by age, sex, and education.
Shang *et al.*, 2020, China^(53)^	Random forest, gradient boost machine	Foods were categorised into twenty-six food groups (% energy/d)	The components from baseline dietary intake served as inputs into machine learning algorithms to determine the most important components for changes in cardiometabolic disease risk. The most important components were then summed to create a Healthy Diet Score.	Changes in cardiometabolic risk were evaluated by subtracting the results at baseline from those at follow-up. The Healthy Diet Score was then tested in an external dataset.	Regression models adjusted for age, sex, household income, parental education.
Wright *et al.*, 2020, USA^(54)^	Treelet transform	Food and Nutrient Database for Dietary Studies food groups were used as inputs.	Eight treelet components (dietary patterns) were identified that explained substantial variation. 1: diets high in salads, moderate vegetables, poultry seafood, fruit, water, tea. 2: loaded on milk, ready-to-eat cereal, bananas. 3: loaded on bread, luncheon meat, table fat, coffee, sugar. 4: loaded on fruit juice, fruit-flavoured drinks. 5: low intake bread, meat, table fats, high intake coffee and sugar. 6: loaded on beef, potatoes, beans, corn. 7: loaded on candy, crackers, pretzels, low-calorie carbonated soft drinks. 8: loaded on pancakes, eggs, French toast.	Quantile regression was used to estimate associations between dietary patterns and periodontitis.	Adjusted models for sex, ethnicity, age, education, smoking status.
Wu *et al.*, 2019, Mexico^(40)^	LASSO	From a 116-item FFQ among adolescents and 104-item FFQ among pregnant women, 21 dietary components were identified as potential methyl-donor rich foods for inclusion in LASSO models.	Seven food items were retained from LASSO models for the maternal diet and eight from the adolescent diet. An Epigenetics Associated Diet Score was created for methyl donor-rich foods, with weights for dietary components obtained from LASSO.	Time-to-event analysis with interval-censored regression models was used to investigate the relationship between Epigenetics Associated Diet Scores and pubertal timing.	Final models adjusted for household socio-economic status (included material wellbeing, highest education level in household).
Xia *et al.*, 2020, China^(55)^	Mutual information	100-item FFQ condensed to twenty-five food groups (g/d) based on similar characteristics.	Dietary patterns were identified using mutual information, identifying five primary dietary components in the cases and six in the controls.	Separate networks were developed for non-alcoholic fatty liver disease cases and controls and were compared.	Case–control. Used propensity score matching to match cases and controls. This process considered sex, age, education, income, smoking status, employment status. Cases that could not be matched closely enough were not included in analyses.
Zhao *et al.*, 2021, USA^(56)^	Bayesian kernel machine regression	FFQ items were assigned to 166 food groups and were further grouped to twelve dietary factors based on evidence of associations with cardiovascular disease.	Bayesian kernel machine regression simultaneously modelled the relationship between the twelve dietary components of interest and cardiovascular disease risk, including the change in each dietary component and disease risk and the dose-response relationship of each dietary component.	Bayesian kernel machine regression was used to jointly consider the 12 dietary components and cardiovascular disease risk.	Balanced recruitment by socio-demographic characteristics. 10-year CVD calculation considers age, sex, race/ethnicity, smoking status. Additionally adjusted regression models for income and education.
***Studies that characterised dietary patterns (no health outcomes/risk)***					
Affret *et al.*, 2017, France^(41)^	Latent class analysis	Created fifty-seven food groups from the 208 food items available in the FFQ.	Used latent class analysis to obtain distinct dietary patterns from these groups, deriving a ‘Healthy’ and ‘Less Healthy’ pattern.	No	Used logistic regression to assess the individual and socioeconomic factors associated with ‘healthy diet’. These factors included education, employment status, income, number of children, marital status, place of residence, size of city, deprivation index, geographical location of birth.
Bezerra *et al.*, 2018, Brazil^(42)^	Latent Class Analysis	A 114-item FFQ was condensed to create thirteen mutually exclusive groups based on nutritional content and level of processing (cereals and tubers and roots; skimmed milk and dairy products; white meats and fish; fruit; vegetables; legumes and nuts; whole milk and dairy products; red meats; processed meats; ultra-processed products; soft drinks and industrialised fruit drinks; sweets; coffee).	Latent class models identified dietary patterns for the whole sample and each generation. Traditionalists: mixed pattern, restricted pattern, prudent pattern. Baby Boomers: mixed pattern, processed pattern, prudent pattern. Generation X: mixed pattern, processed pattern, prudent pattern.	No	Assessed how dietary patterns differed by three birth cohorts (age).
De Almeida *et al.*, 2022, Brazil^(57)^	Treelet transform	114-item FFQ condensed to twenty-five food groups based on nutritional similarity.	Used three analytic approaches to derive dietary patterns (treelet transform, factor analysis, reduced rank regression). Treelet transform identified three patterns: convenience, prudent, and rice and beans. Factor analysis identified three patterns: Convenience, Brazilian traditional, and prudent. Reduced rank regression identified three patterns but only retained one, the convenience pattern.	No	Assessed how dietary patterns differed by socio-demographic characteristics: age, sex, smoking status, education, race
Farmer *et al.*, 2020, USA^(58)^	Latent class profile analysis	11 out of 12 components from the Healthy Eating Index-2010 served as inputs (1. total vegetable, 2. greens/beans, 3. total fruit, 4. whole grains, 5. dairy, 6. total protein foods, 7. seafood and plant proteins, 8. fatty acid ratio, 9. sodium, 10. refined grains, 11. solid fats, alcohol and added sugars).	Distinct classes for total day intake and dinner intake were identified. Classes included (total day intake): Standard American Diet, Standard American Diet with low Na, Healthy USA, Healthy USA with high vegetable, Healthy USA low sodium.	No	Multinomial logistic regression used to assess how latent classes differed by socio-demographic characteristics, including age, sex/gender, race/ethnicity, marital status, education, employment status, poverty/income ratio, number of people in household.
Harrington *et al.*, 2014, Republic of Ireland^(43)^	Latent class analysis	Food items were aggregated from a 159-item and 167-item FFQ into twenty-three mutually exclusive food groups (g/d, ml/d)	Latent class analysis identified three dietary patterns: Western, Healthy, and low-energy.	No	Assessed relationships between latent classes and socio-demographic characteristics, including smoking status, education, marital status.
Hearty & Gibney, 2008, Republic of Ireland & Northern Ireland^(44)^	Artificial neural networks and decision trees	Food codes were aggregated to sixty-two food groups.	Food groups were provided to neural networks and decision trees to identify dietary patterns predictive of diet quality quintile by meal.	No	No
Iqbal *et al.*, 2016, Germany^(45)^	Gaussian graphical models (GGM); confirmed with Semiparametric Gaussian Copula graphical models	Items from the 148-item FFQ were grouped into forty-nine groupings and were log-transformed.	GGM were applied to derive dietary patterns among males and females. Edges represented conditional dependencies between food groups. Food groups that were not connected by an edge were conditionally independent.	No	Identified sex-specific graphical networks.
Schwedhelm *et al.*, 2018, Germany^(46)^	Semiparametric Gaussian Copula graphical models	Food codes were condensed to thirty-nine food groups.	Semiparametric Gaussian Copula graphical models derived meal-specific networks. For example, the breakfast network was characterised by one primary network with five communities and included foods such as breakfast cereals, margarine, and eggs. Edges represented conditional dependencies between food groups. Food groups that were not connected by an edge were conditionally independent.	No	No
Schwedhelm *et al.*, 2021, USA^(59)^	Gaussian graphical model	Foods were grouped into forty food groups based on the Food and Nutrient Database for Dietary Studies.	Diet quality was assessed using the Healthy Eating Index 2015, and participants were split into tertiles of HEI 2015 scores, to compare dietary patterns by meal of women in the low and high HEI tertile generated using Gaussian graphical models. Edges represented conditional dependencies between food groups. Food groups that were not connected by an edge were conditionally independent.	No	No
Solans *et al.*, 2019, Spain^(47)^	3 Compositional data analysis approaches: compositional PC analysis, balances, principal balances	Nine items (vegetables, fruit, legumes, seafood, cereals, meat, dairy, monounsaturated fats, and saturated fats) from the 140-item FFQ were included as inputs.	Three variations of compositional data analysis were used to explain the variance among the dietary components and identify dietary patterns.	No	No

* We considered socio-demographic characteristics that are related to social position or are indicators of equity including age, sex, gender, race/ethnicity, marital status, education, employment status, smoking status as examples. We did not include physical activity, BMI or alcohol consumption.

**Table 4.** Novel methods applied to identify dietary patterns across included studies^*^

Method of analysis	Description	References
Methods with applications in machine learning		
Classification models	Algorithms that can be used for prediction and classification^(76,77)^.	
Random forest	The random forest is generated from a wide array of trees that are randomly sampled and averaged. Random forest models can additionally be used on regressors^(76)^.	(Biesbroek *et al.* 2015; Shang *et al.* 2020)
Decision tree	Trees include nodes (variables) and branches, representing possible outcomes from a node. This forms an ‘inverted tree’, beginning with a root node and branching to many possible end nodes^(77)^.	(Hearty and Gibney 2008)
Neural network	The structure of this algorithm was based on the human brain and is well suited to complex, non-linear problems^(78)^.	
Artificial neural network	The algorithm starts with input nodes, which communicate, forming hidden layers of connections within the network, and provide learned output^(78)^.	(Hearty and Gibney 2008)
Kohonen neural network	A neural network comprised of a single layer. Data enter through input nodes and are organised to a simple structure of output nodes^(79)^.	(Jamiołkowski, Szpak, and Pawłowska 2005)
Probabilistic graphical models	Algorithm that can be used to determine joint relationships between nodes, forming a graphical network, where connections between nodes are represented by edges. These connections may be full- or partial-order correlations^(80–82)^.	
Gaussian graphical models	Probabilistic graphical model with data following a Gaussian distribution^(81)^.	(Hoang, Lee, and Kim 2021; Iqbal *et al.* 2016; Schwedhelm *et al.* 2021)
Mixed graphical models	Probabilistic graphical model with mixed data (i.e. Gaussian and categorical)^(81)^.	(Hoang, Lee, and Kim 2021)
Semiparametric Gaussian Copula graphical models	Probabilistic graphical model with data not required to be normally distributed^(83)^.	(Iqbal *et al.* 2016; Schwedhelm *et al.* 2018)
Other machine learning approaches		
Gradient boost machine	Machine learning algorithm that applies a boosting ensemble method to decision trees. This allows a weak learning algorithm to provide predictions through weighted averages of weak predictions^(53,84)^.	(Shang *et al.* 2020)
Bayesian kernel machine regression	Machine learning algorithm that uses a kernel function to consider the effects of multiple exposures, covariates, and confounders on an outcome^(85)^.	(Zhao *et al.* 2021)
Compositional data analysis	Method that identifies the relative magnitude of differences between variables to determine relative importance^(86,87)^.	(Solans *et al.* 2019)
Least Absolute Shrinkage and Selection Operator (LASSO)	A method that selects the most important variables and reduces the number of variables in the model^(88)^.	(Wu *et al.* 2019)
Multivariate finite mixture models		
Latent Class Analysis	A method that creates mutually exclusive groups (classes) of participants based on similar latent characteristics^(41,42)^.	(Affret *et al.* 2017; Bezerra *et al.* 2018; Dalmartello *et al.* 2020; Harrington *et al.* 2014; Hose *et al.* 2021)
Latent class transition model	Allows for transition between identified groups^(51,89)^.	(Madeira *et al.* 2021)
Latent class profile analysis	Latent class analysis when continuous variables are used^(58)^.	(Farmer *et al.* 2020)
Multivariate finite mixture models	Identifies mutually exclusive groupings where cluster shape is not limited^(90)^.	(Fonseca *et al.* 2012; Oliveira *et al.* 2011)
Mutual information	This method creates networks, estimating the amount that uncertainty is reduced in one variable by knowing another^(91)^.	(Samieri *et al.* 2020; Xia *et al.* 2020)
Treelet transform	Data-driven approach that reduces dimensionality and is a hybrid between hierarchical clustering and principal component analysis^(92,93)^.	(de Almeida Alves *et al.* 2022; Wright *et al.* 2020)

* Studies may have used more than one novel approach to characterise dietary patterns.

In twelve studies, two to eight distinct dietary patterns, such as the ‘prudent’ pattern or ‘Western’ pattern, were identified using methods such as latent class analysis, treelet transform, random forest with classification tree analysis and multivariate finite mixture models^(36–38,41–43,48,50,51,54,57,58)^. Six studies applied network methods, including probabilistic graphical models and mutual information, to identify networks of dietary patterns among populations^(45,46,49,52,55,59)^.

Dynamism, or how dietary patterns vary across time, was incorporated into four studies’ characterisation or analysis of dietary patterns. Three studies incorporated stratification by meals to consider dynamism^(44,46,59)^. In two studies using graphical models, separate networks were created for each meal to provide insights into how patterns of intake vary throughout the day^(46,59)^. Hearty and Gibney used decision trees and neural networks and ran models by meals based on sixty-two food groups to predict diet quality^(44)^. Additionally, one study considered dynamism by using ANOVA and chi-square tests to descriptively show how a variety of characteristics were associated with stable or changing dietary patterns characterised using latent class analysis^(43)^.

Fourteen studies examined relationships between dietary patterns characterised using novel methods and variables indicative of health risk or outcomes, such as periodontitis, cardiovascular disease and metabolic syndrome (Table 3) ^(36–40,48–56)^. Six studies included longitudinal analysis of the relationship between dietary patterns and health outcomes^(36,38,40,50,52,53)^. Most studies that examined health risk or outcomes first identified dietary patterns using a novel method and then investigated relationships with health outcomes using regression models^(36–40,48,50,51,54)^. In contrast, some studies incorporated variables indicative of health outcomes or risk directly into the machine learning models^(49,56)^. For example, Zhao *et al.* ^(56)^ applied Bayesian kernel machine regression, a machine learning model designed to incorporate high-dimensional data, to jointly model the relationship between several dietary components and cardiovascular disease risk. Similarly, Hoang *et al.* ^(49)^ included health variables within mixed graphical models, though directionality of diet-health relationships could not be ascertained given the cross-sectional nature of the data. In two case–control studies, dietary patterns were identified using mutual information to estimate dietary pattern networks, with stratification by health outcomes^(52,55)^.

Nineteen studies considered socio-demographic characteristics, such as sex, age, race/ethnicity, education and income^(36,37,39–43,45,48–58)^. In one case, socio-demographic characteristics were included in models used to characterise dietary patterns^(49)^. Two studies stratified by socio-demographic characteristics, examining dietary patterns by sex^(45)^ or age groups^(42)^. Studies that used case–control designs typically considered socio-demographic characteristics through matching^(52,55)^. In the remaining studies that considered socio-demographic characteristics, these were incorporated in regression models to explore how dietary patterns characterised using novel methods were associated with health and other characteristics.

Two studies included comparisons of novel methods and traditional statistical approaches^(36,57)^. For instance, Biesbroek *et al.* ^(36)^ found that dietary patterns identified through reduced rank regression were more strongly associated with coronary artery disease compared with those identified through random forest with classification tree analysis.

## Discussion

The application of novel methods to dietary pattern research is rapidly expanding, with the aim of better understanding their complexity and how they are related to health and other factors. Many studies used methods that characterise distinct dietary patterns based on the population being studied, such as the ‘prudent’ pattern or the ‘Western’ pattern. Most studies used cross-sectional data, limiting opportunities to examine the effect of dietary patterns on health.

Methods newly being applied in this field offer promising capacity to better understand the totality of dietary patterns and synergistic relationships among dietary components when compared with traditional approaches that do not assume synergy^(4,14)^. Given the large variation in how dietary patterns were characterised using novel methods, multidimensionality and potential synergistic relationships between dietary components were considered and presented in a range of ways, from latent classes to networks. Several studies incorporated dynamism into their consideration of dietary patterns, though in most cases this was through stratification, for example, by meal, rather than through direct use of novel methods^(43,44,46,59)^. In these cases, it was a combination of input variables, stratification by time and the novel method that enabled explorations of dynamism.

The methods highlighted have a range of strengths and limitations for the characterisation of dietary patterns. Methods that focused on the classification of distinct patterns allowed for the assessment of relationships between these patterns and health outcomes or other indicators of interest but explored the interrelationships between dietary components to a lesser degree^(36–38,41–43,48,50,51,54,57,58)^. Other methods such as compositional data analysis, mutual information and probabilistic graphical models can be used to consider joint relationships among dietary components to better understand multidimensionality^(45–47,49,52,55,59)^. For example, Gaussian graphical models provide the opportunity to visualise the dietary pattern through a network of dietary components, with relationships between variables indicating conditional dependencies^(45,49,59)^. However, studies making use of these methods included further analyses, such as the development of a score from dietary pattern networks, to assess relationships with health outcomes.

There are trade-offs between novel and traditional methods that should be considered when contemplating the most appropriate methods for a given study. Though potential benefits such as a greater ability to discern multidimensionality may be desirable, these must be weighed against the implications for interpretability and computational costs. The application of novel methods may not always yield insights beyond those gained from traditional approaches. For example, Biesbroek *et al.* ^(36)^ found that random forest models did not outperform reduced rank regression when examining associations of dietary patterns with coronary artery disease. Conversely, a study that was not included in this review because it first identified dietary patterns using a traditional method – principal component analysis – found that machine learning algorithms were better able to classify the identified dietary patterns according to cardiometabolic risk compared to traditional approaches^(60)^.

Several socio-demographic characteristics are indicators of systemic health inequity and have been shown to be associated with dietary patterns among populations^(61–63)^. The degree to which studies incorporated socio-demographic characteristics into their consideration of dietary patterns or relationships between dietary patterns and health varied, with adjusted regression models applied after dietary patterns were characterised as the most common approach. Consistent with nutrition research more broadly^(63–65)^, there was little consideration of possible interactions among socio-demographic characteristics in relation to dietary patterns. Methods particularly suited to pattern recognition and complexity could be leveraged to simultaneously explore potential joint relationships among facets of social identity and dietary patterns^(66)^ and advance our understanding of how broader systems of oppression and intersecting characteristics contribute to dietary patterns^(62)^.

Beyond the inclusion of socio-demographic characteristics in models, considering equity from the beginning of study design is a critical consideration given potential bias in data and algorithms that can have immense implications for those who already experience inequities because of factors such as structural racism^(67–69)^. The included studies did not explicitly discuss the incorporation of equity into study design, and many conducted secondary analyses of existing datasets. The use of directed acyclic graphs has been identified as a potential solution to mitigate some possible issues with bias through careful model design^(67)^ and has been applied in other domains of nutrition research using novel methods^(14)^. Engaging individuals with lived experience and the integration of interdisciplinary teams with broad expertise that can combine content knowledge with data-driven approaches can help to mitigate potential bias in algorithms^(66)^.

The level of description of methods varied, and it was sometimes challenging to decipher the specifics of how novel methods were applied. Although the Strengthening the Reporting of Observational Studies in Epidemiology—Nutritional Epidemiology reporting guidelines provide guidance for transparently reporting nutritional epidemiology and dietary assessment research^(70)^, it was not designed specifically for the methods used in the studies considered in this review and the ways in which they are being applied in dietary patterns research. Other reporting guidelines, such as the Consolidated Standards of Reporting Trials, have been extended to consider the application of artificial intelligence (AI) ^(71)^. Motivations related to the extension of Consolidated Standards of Reporting Trials included inadequate reporting of studies using AI and the lack of full consideration of potential sources of bias specific to AI within existing reporting guidelines^(71)^. Relevant items added to Consolidated Standards of Reporting Trials-AI pertain to the role of AI in the study, the nature of the data used in AI systems and how humans interacted with AI systems, for example^(71)^. The extension of reporting guidelines such as STROBE-nut to consider applications of AI, including machine learning, and other methods that are becoming more commonly used, may facilitate consistent and complete reporting and improved comparability of studies. Reporting guidelines should continue to emphasise strategies applied to mitigate measurement error in dietary intake data^(70)^, as studies using novel methods are not immune to the effects of error on findings^(72)^. Along with reporting guidelines, the development of tailored quality appraisal tools may facilitate synthesis of high-quality evidence to inform recommendations about dietary patterns and health.

This review provides a snapshot of a rapidly evolving field^(73,74)^, with the involvement of an interdisciplinary team of researchers lending to a robust consideration of emerging methods in dietary patterns research. While prior reviews have provided perspectives on the potential applications of machine learning within the field of nutrition^(19–21)^, this review considered dietary patterns in particular, as well as considering approaches beyond machine learning that have not traditionally been used in this area, broadening the scope compared to prior reviews^(22,75)^. The search terms were informed by preliminary searching, though it is unlikely that all relevant articles applying novel methods to characterise dietary patterns were captured, partially driven by the wide range of descriptors used for these methods and the lack of reporting standards. As well, determining whether a method is novel is somewhat subjective. Methods such as factor analysis and principal component analysis once revolutionised dietary pattern analysis, providing data-driven approaches to identify patterns^(15)^. Now, they are widely applied and recognised as limited in their capabilities to capture complexity compared to some newer approaches. Further, the search terms skewed toward multidimensionality *v*. dynamism, potentially overlooking some studies focusing on variation of dietary patterns over time or across eating occasions. Nonetheless, this review documents an acceleration of the application of a range of novel methods to dietary patterns research and captures a broad scope of methods being used to characterise these patterns, highlighting the need for researchers to develop the lexicon and knowledge needed to interpret the emerging literature.

### Conclusion

The findings of this review indicate a strong motivation to apply novel methods, including but not limited to machine learning, to improve understanding of dietary patterns and how they relate to health and other factors. The application of these methods may help us to learn about complex relationships that may not be possible to discern through traditional approaches. However, these methods may not be suitable for every question and do not necessarily overcome the limitations of more traditional approaches.

Given the proliferation of these methods, it is becoming increasingly worthwhile for nutrition researchers to have at least a basic understanding of novel methods such as machine learning and latent class analysis, so they can interpret the results of emerging studies. The development and implementation of reporting guidelines and quality appraisal mechanisms for studies that apply novel methods may improve the capacity for synthesis of evidence generated to inform strategies that promote improved population health and well-being.

## Supporting information

Hutchinson et al. supplementary material 1 Hutchinson et al. supplementary material

Hutchinson et al. supplementary material 2 Hutchinson et al. supplementary material

## Supplementary material

For supplementary material/s referred to in this article, please visit https://doi.org/10.1017/S0007114524002587
