# Supplementary material for: Advances in methods for characterising dietary patterns: a scoping review
Source: Br J Nutr. 2025 Mar 10;133(7):987–1001. doi: 10.1017/S0007114524002587 (PMC12198347; doi:10.1017/S0007114524002587)
Supplement: Hutchinson et al. supplementary material 2 — Hutchinson et al. supplementary material [file S0007114524002587sup002.pdf]

Supplemental File 2: Key extraction fields for review of novel analytic methods to characterize dietary patterns

Field	Description
Study identification	Authors
	Title
	Journal
	Year published
	Funding source for study
Study population	Sample size
	Sample characteristics
Methods	Research objectives/questions
	Study design
	Study name
	Measurement of diet
	Dietary components analyzed
	Novel methods of analysis
	Consideration of equity
Results	Findings re: diet or diet-related outcomes
	Author description of method utility and implications
Critical review	Author stated limitations
	Reviewer observed limitations
